# Neurovascular Unit Dysfunction and Neural Barrier Pathology in Dysautonomia: Molecular Links to Cardiovascular Autonomic Failure

**DOI:** 10.3390/ijms27188375

**Published:** 2026-09-20

**Authors:** Bernard Kordas, Kamila Zglejc-Waszak, Patryk Mizia, Wojciech Matuszewski, Agnieszka Skowrońska, Mariusz Majewski, Judyta K. Juranek

**Affiliations:** 1 Department of Human Physiology and Pathophysiology, School of Medicine, Collegium Medicum, University of Warmia and Mazury in Olsztyn, 10-082 Olsztyn, Poland; 2 Department of Anatomy and Histology, School of Medicine, Collegium Medicum, University of Warmia and Mazury in Olsztyn, 10-082 Olsztyn, Poland; 3 Clinic of Endocrinology, Diabetology and Internal Medicine, School of Medicine, Collegium Medicum, University of Warmia and Mazury in Olsztyn, 10-561 Olsztyn, Poland

**Keywords:** neurovascular unit, blood–brain barrier, blood–nerve barrier, dysautonomia, cardiovascular autonomic neuropathy, hypertension, heart failure, neuroinflammation

## Abstract

Cardiovascular autonomic failure arises from lesions across central nuclei, peripheral nerves, and autonomic ganglia. This review examines whether dysfunction of the neurovascular unit and neural barriers contributes to that failure. The clearest evidence comes from rodent models of hypertension and heart failure. Increased permeability in the PVN, NTS, and RVLM develops alongside sympathetic activation and impaired baroreflex control. Angiotensin II and inflammation appear to affect endothelial transport and junctional integrity. Exercise and interventions directed at these pathways improve barrier function together with autonomic control. Peripheral evidence is less direct. Diabetes and inflammatory neuritis alter the blood–nerve barrier, but most experiments examine somatic or sensory nerves and do not establish causation in cardiovascular autonomic neuropathy. Autonomic ganglia also differ in normal vascular permeability, which precludes treating them as uniform extensions of the blood–nerve barrier. Human studies show altered barrier measurements in heart failure and synucleinopathies, yet rarely pair regional permeability with validated autonomic tests. Neural barrier dysfunction may contribute to dysautonomia in defined anatomical and clinical settings. Its importance remains uncertain outside the pathways examined directly, and future studies should relate regional barrier changes to autonomic function over time.

## 1. Introduction

Dysautonomia describes impaired autonomic control that can arise at different levels of the nervous system. Its cardiovascular presentation depends on the affected pathway and may include impaired cardiac regulation, orthostatic hypotension, or excessive sympathetic activity. The disorders considered here differ substantially in the site and mechanism of autonomic injury. Autonomic dysreflexia after spinal cord injury forms a separate cardiovascular syndrome [1]. A useful mechanistic review must preserve these anatomical differences because the evidence for vascular and barrier involvement is not equally strong at every level of the autonomic nervous system.

The blood–brain barrier (BBB) forms the vascular interface of the neurovascular unit (NVU)—its restrictive properties depend mainly on specialized brain endothelial cells and their interactions with pericytes, astrocytes, and the surrounding neural tissue. Macrophage–endothelial communication is now recognized as a dynamic part of the vascular response to systemic and neural signals [2]. Tight and adherens junctions limit movement between endothelial cells, but barrier function also depends on low vesicular flux, endothelial polarity, influx and efflux systems, and interactions between endothelial cells and adjacent cells [3,4,5,6]. The blood–nerve barrier (BNB) is a distinct peripheral structure. It is formed by endothelial cells of endoneurial microvessels, while the perineurium provides an additional diffusion barrier between the epineurial and endoneurial compartments [7,8,9]. The term NVU should not be used as a synonym for the BNB. Throughout this review, “neural barrier dysfunction” is used only as an umbrella term for abnormalities of anatomically distinct interfaces. The specific terms BBB, endoneurial BNB, perineurium, and autonomic ganglion vasculature are used whenever the assessed structure can be identified; NVU denotes the multicellular central unit and is not interchangeable with any individual barrier.

Interest in neural barriers has often centered on overt leakage caused by loss of junctional proteins. That view is incomplete. Experimental mouse studies show that lipids transported by MFSD2A suppress caveola formation, whereas pericyte deficiency increases endothelial transcytosis [10,11]. Conversely, deletion of claudin-5 in only a subset of adult endothelial cells produces rapid permeability to molecules of restricted size and early glial responses [12]. Endothelial RNA sequencing showed that mouse models of several neurological disorders share a core pattern of BBB dysfunction [13]. This common endothelial response does not make the initiating insults or all molecular changes interchangeable. Barrier failure can thus begin through changes in transcellular transport, paracellular transport, or both, and the route may determine which circulating factors enter neural tissue.

This distinction is especially relevant to cardiovascular autonomic control. Several protected nuclei receive or integrate information from circumventricular structures that normally sample the circulation. A pathological increase in permeability within the protected parenchyma could enlarge the influence of circulating angiotensin II, cytokines, immunoglobulins, or plasma proteins beyond these physiological entry points. The same principle may apply in peripheral nerves, where changes in endoneurial permeability alter ionic balance, fluid pressure, oxygen delivery, and access of immune cells to axons. These mechanisms are plausible, but plausibility alone does not show that a barrier lesion causes dysautonomia.

The present review focuses on the molecular and cellular evidence that most directly connects a neural barrier measurement with a cardiovascular autonomic endpoint. Hypertension and heart failure provide the clearest central evidence, while diabetes provides the most relevant peripheral framework. Immune and neurodegenerative disorders are retained as informative comparisons.

## 2. Methodology

We conducted a structured narrative review of articles published in English between January 1995 and August 2026. Initial exploratory PubMed searches and targeted Scopus searches were used to identify the terminology employed across the relevant disease-specific, anatomical, and mechanistic literature. PubMed searches combined terms for neural barriers, including “neurovascular unit”, “blood–brain barrier”, and “blood–nerve barrier”, with terms describing autonomic dysfunction. The searches were refined by disease, anatomical site, or mechanism according to the subject of each section. The complete PubMed search string is provided in Appendix A. Scopus was used to identify publications indexed under different terminology and to trace citations from key experimental studies. No single predefined Scopus search string was applied.

Original articles in peer reviewed journals were eligible when they provided structural or functional evidence of altered barrier function in a relevant disease or experimental model. Priority was given to studies that assessed a neural barrier and cardiovascular autonomic function in the same experiment. Evidence of barrier dysfunction had to be based on permeability measurements or direct assessment of endothelial structure and transport. Autonomic relevance was judged from physiological measures of cardiovascular control. Study selection and synthesis were qualitative. Search results were deduplicated and screened by title and abstract, followed by full-text assessment of potentially eligible publications. The primary screening was performed by one author; studies retained for the synthesis were checked by two authors, and uncertain eligibility decisions were resolved by another. Records identified through citation tracking were assessed using the same criteria.

Human studies, animal experiments, and cell models were considered. Review articles were included selectively to define established anatomy, terminology, clinical context, or areas in which primary evidence was sparse. Studies of sensory or motor nerves and nonautonomic brain regions were retained only when they described mechanisms not yet examined directly in autonomic structures; these findings were identified as extrapolations. Studies limited to systemic endothelial dysfunction without a relevant neural or autonomic component, generalized inflammation without an assessment of a neural barrier or vascular transport, and peripheral neuropathy without evidence concerning the BNB, perineurium, or ganglion vasculature were excluded. Conference abstracts, editorials, protocols, and isolated case reports were not considered.

The review includes 74 publications. Evidence was interpreted according to the species and experimental model, anatomical location, directness of the barrier measurement, presence of an autonomic outcome, temporal relation between barrier and autonomic changes, and response to intervention. Evidence was classified as direct when a barrier measure in a defined autonomic structure and a cardiovascular autonomic outcome were obtained in the same study. Evidence was classified as indirect when autonomic relevance was inferred from another anatomical site or model or from a separate clinical association. Evidence was considered limited when either the barrier or autonomic domain was absent or assessed using a nonspecific proxy, and strongest when direct regional measurements were supported by temporal, interventional, or mechanistic data. These descriptors indicate relevance to the review question and do not constitute a formal risk-of-bias assessment. No formal risk of bias instrument or quantitative synthesis was applied because the article was designed as a narrative mechanistic review.

## 3. Neural Barrier Organization in Cardiovascular Autonomic Pathways

The central autonomic network includes cortical and subcortical structures, but arterial pressure and cardiorespiratory reflexes depend particularly on hypothalamic and medullary nodes—the paraventricular nucleus of the hypothalamus (PVN) integrates neuroendocrine signals with sympathetic and parasympathetic output; the nucleus tractus solitarius (NTS) receives baroreceptor and visceral afferent input, while the rostral ventrolateral medulla (RVLM) provides a major excitatory drive to sympathetic preganglionic neurons [14,15,16]. These nuclei normally contain continuous, nonfenestrated microvessels. The extracellular environment of these nuclei remains separated from the circulation, despite nearby circumventricular organs that normally detect circulating signals. Endothelial junctions alone do not provide this protection. Controlled transcytosis and communication between vascular, glial, and immune cells also maintain barrier function. These components can change independently. An increase in small tracer leakage, for example, does not establish unrestricted entry of immunoglobulins or cells. Likewise, reduced claudin-5 staining does not quantify functional permeability unless it is paired with a tracer or another transport measurement. Regional leakage is most informative when interpreted together with structural and physiological data. In peripheral nerves, endoneurial capillaries differ from epineurial vessels in their junctions and transport systems. RNA sequencing of cultured human endothelial cells and sural nerve microvessels defined this molecular profile, which was validated in four histologically normal adult nerves [8,17]. The perineurium adds another level of metabolic and diffusion control [7,18]. The endoneurial vasculature and perineurium maintain an environment that supports axonal conduction [9]. Autonomic axons share this environment when they travel within mixed nerves, although most human studies of the BNB use somatic nerve samples and do not examine autonomic fascicles.

Autonomic ganglia require separate consideration. Classical tracer studies reached different conclusions about a uniform blood–ganglion barrier. A broad comparison found substantially greater access of circulating proteins to sensory and sympathetic ganglia than to nerve trunks, while ultrastructural work showed that permeability varies between sympathetic ganglia and even between vascular territories within one ganglion [19,20]. Capillaries around principal neurons in superior cervical and thoracic ganglia can restrict horseradish peroxidase, whereas regions containing small granule cells and parts of the coeliac–mesenteric complex permit greater entry of smaller tracers [20]. Species, ganglion, tracer size, and endothelial phenotype all matter. It is consequently inaccurate to assume that pathogenic antibodies can reach autonomic ganglia only after a conventional BNB has broken down. Thus, circulating antibodies or inflammatory mediators may enter normally permissive vascular territories without acquired barrier failure, whereas access to restrictive territories may require local endothelial change. This question must therefore be assessed separately for each ganglion, vascular region, and molecular size.

The dorsal root ganglion provides a modern example of regional heterogeneity, although it is sensory and should not be treated as an autonomic ganglion. Reviews of peripheral neural interfaces distinguish the restrictive endoneurial BNB from the more permeable vascular environment of dorsal root ganglia [21]. Mouse studies show that permeability changes along the vascular tree of the dorsal root ganglion: arterial endothelium is restrictive, while venous segments are more permeable. CD163-positive perivascular macrophages sample macromolecules entering from the circulation through caveolae. Both features were also found in human dorsal root ganglion tissue [22]. Although visceral afferents in this ganglion take part in autonomic reflexes, equivalent vascular organization has not been demonstrated in sympathetic or parasympathetic ganglia. They show why anatomical labels cannot be substituted for direct barrier measurements.

A final distinction concerns the direction of causality. Barrier change may precede altered autonomic activity, arise from sustained hypertension or systemic inflammation, or persist after neural injury has begun. It may also be compensatory, for example by permitting removal of debris or entry of protective immune mediators. Temporal studies, interventions that alter the barrier without directly changing neuronal excitability, and rescue experiments are required to separate these possibilities. This standard is met only in part by the current literature.

**Figure 1:**
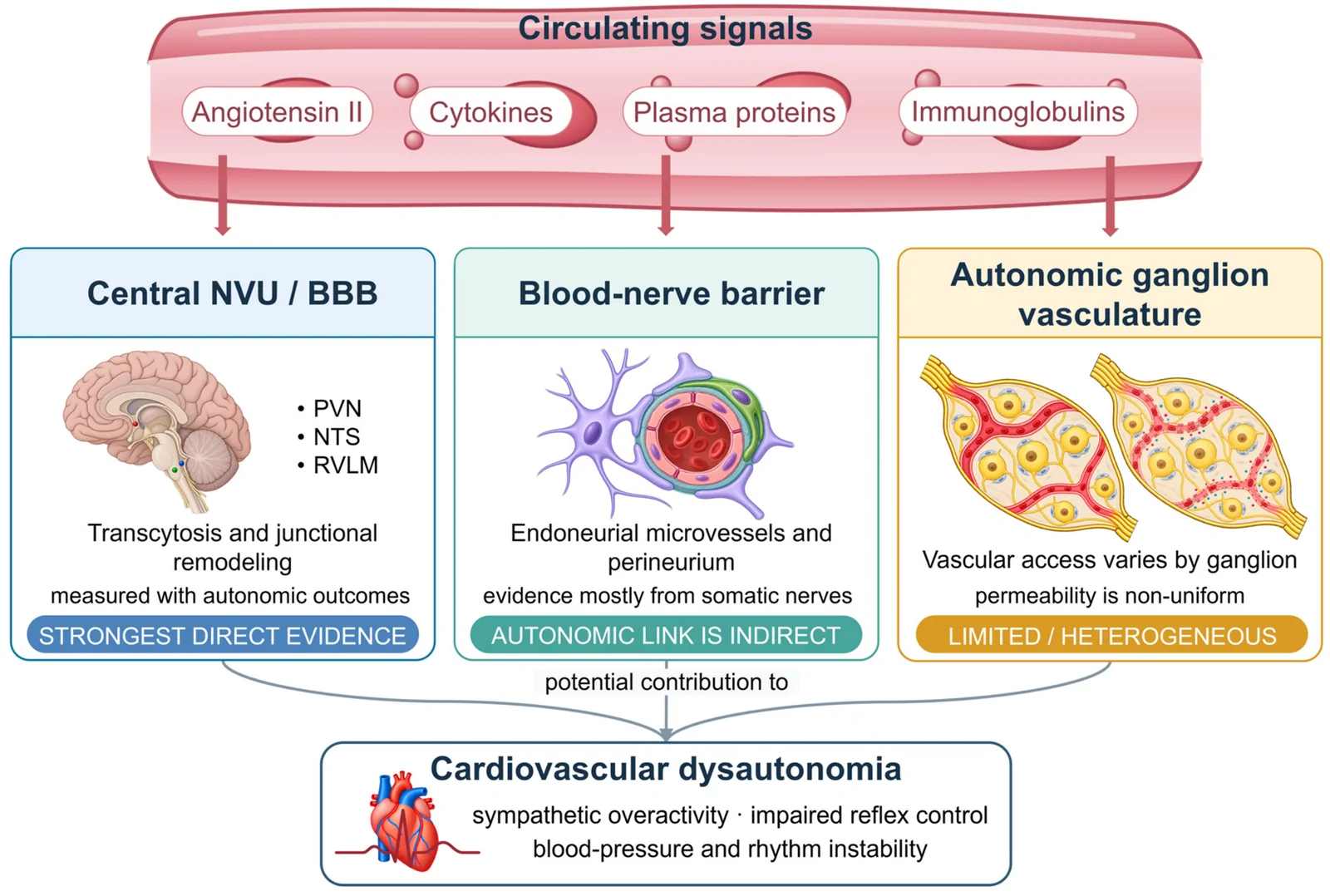
Neural barrier interfaces potentially involved in cardiovascular dysautonomia. Central BBB dysfunction in the PVN, NTS, and RVLM has the strongest direct experimental support, whereas evidence for the BNB is mostly indirect and autonomic ganglia show heterogeneous vascular permeability. These interfaces may contribute to sympathetic overactivity, impaired reflex control, and cardiovascular instability. Abbreviations: BBB, blood–brain barrier; BNB, blood–nerve barrier; NTS, nucleus tractus solitarius; PVN, paraventricular nucleus of the hypothalamus; RVLM, rostral ventrolateral medulla.

The anatomical organization of these neural interfaces and the relative strength of the evidence linking their dysfunction to cardiovascular autonomic abnormalities are summarized in Figure 1.

## 4. Central Barrier Dysfunction in Hypertension and Heart Failure

The most coherent evidence begins with experimental hypertension. In spontaneously hypertensive rats, tracer leakage is greater in the PVN, NTS, and RVLM than in normotensive controls. Changes in barrier proteins accompany this leakage, and circulating angiotensin II is found outside vessels in proximity to neurons and microglia. Losartan prevents these changes, while hydralazine lowers arterial pressure without the same protection of the BBB. This comparison argues that signaling through the angiotensin II type 1 receptor, and not elevated pressure alone, contributes to the barrier lesion [23]. It also establishes a route by which a circulating peptide can reach nuclei that regulate sympathetic tone.

Perivascular macrophages and endothelial cells participate in this process. In an angiotensin II-infused mouse model, endothelial angiotensin II type 1 receptors initiated BBB disruption, while angiotensin II type 1 receptor–NOX2 signaling in perivascular macrophages was required for its full expression. Brain macrophage depletion or deletion of the receptor in perivascular macrophages partially reduced permeability; downregulation of the endothelial receptor prevented BBB disruption [24]. These findings place the vascular interface upstream of at least part of the neural response. They do not show that the macrophage manipulations reduced the hypertensive response, and they do not imply that all microglial or macrophage activation follows barrier opening, because angiotensin II and cytokines can influence central autonomic circuits through circumventricular organs and neural pathways as well.

The interaction between inflammation and barrier function has been tested more directly in spontaneously hypertensive rats. TLR4 inhibition also suppresses microglial activation and lowers the expression of IL-6 and TNF-α. The pressor response to ganglionic blockade is attenuated. AT1 receptor inhibition produces similar changes and normalizes arterial pressure [25]. Since both treatments affect several cell types, these experiments do not identify the endothelium as their principal site of action. The concurrent improvement in permeability and autonomic function still supports a link between the processes. A later mouse study implicated interactions between kinin B1 and AT1 receptors during angiotensin II infusion. Inhibiting the kinin receptor improved autonomic function and reduced microglial activation in the PVN, although permeability was not measured [26].

Longitudinal observations refine the sequence. During the transition from a prehypertensive state to established hypertension, local angiotensin II signaling rises before clear tracer leakage. Increased permeability and microglial activation then appear in the PVN, NTS, and RVLM as pressure and autonomic abnormalities develop [27]. This timing is compatible with angiotensin II initiating changes at the vascular and glial interface, but it does not show that leakage is indispensable for the first increase in blood pressure. Barrier dysfunction is better viewed as an amplifier that helps maintain access of circulating and perivascular signals to central autonomic circuits.

Endothelial transcytosis appears especially important in the PVN. In hypertensive rats, capillaries show increased caveolin-1 and tracer leakage consistent with increased vesicular transport, without a parallel fall in claudin-5. Four weeks of aerobic training reduces tracer extravasation and caveolin-1 expression while autonomic indices improve [28]. Earlier work also found that exercise reduces leakage in the PVN, NTS, and RVLM and improves baroreflex and sympathetic measures even when hypertension persists [29]. These results correct the assumption that a leaky BBB necessarily reflects widespread dissolution of tight junctions. They also provide an intervention in which improved autonomic control occurs together with normalization of a defined transport route.

Exercise has effects beyond the endothelium. In the PVN of hypertensive rats, training reduces HMGB1, inflammatory cytokines, and microglial activation while restoring autonomic balance [30]. Results from related models indicate that exercise affects both barrier integrity and inflammation, although their temporal order remains unclear. Reviews of exercise studies also show that BBB responses vary with the protocol and disease model [31,32]. The IL-17 axis may contribute to this interaction. In rats infused with angiotensin II, RORγt inhibition preserved MFSD2A and limited caveolin-1 expression and tracer leakage in PVN microvessels. This was accompanied by less microglial activation and by lower sympathetic tone and arterial pressure [33].

Heart failure provides a second, closely linked model. Coronary ligation in rats increases BBB permeability in the PVN, NTS, and RVLM. This is accompanied by stronger sympathetic drive and loss of parasympathetic and reflex control. Endothelial cells contain more vesicles and their junctional contacts are shorter; caveolin-1 expression rises and claudin-5 falls. Exercise reverses these barrier abnormalities and improves autonomic function despite persistent cardiac injury [34]. This study offers unusually direct alignment of anatomy, ultrastructure, permeability, and physiology.

Later experiments in the same heart failure model found that exercise and losartan produced similar changes. Each lowered angiotensin II in the PVN and reduced both vesicular transport and junctional damage. Leakage and microglial activation declined, while sympathetic drive decreased and cardiac parasympathetic control improved. Combining the treatments offered no clear additional benefit [35]. This convergence supports the involvement of angiotensin II but leaves the responsible cell population unresolved.

IL-17A provides another connection between systemic inflammation and the PVN in heart failure. After myocardial infarction in male Sprague–Dawley rats, a 10 kDa tracer leaked into the PVN but not into the sampled cortex. The amount of leakage correlated with plasma IL-17A. Local knockdown of IL-17 receptor A reduced permeability. Caveolin-1 expression fell, while proteins associated with tight junctions were restored [36]. The regional comparison and local intervention strengthen the inference that an inflammatory signal acts at the endothelial interface of an autonomic nucleus.

Human evidence in heart failure is more limited and uses different measurements. A diffusion-weighted arterial spin labeling study compared 27 adults with heart failure with 59 controls. Water exchange and arterial transit time differed between the groups across several cortical and subcortical regions. The reported correlations concerned cognition and mood, and the study did not pair imaging with formal autonomic testing. Water exchange also differs from macromolecular tracer leakage. The findings support altered BBB physiology in human heart failure, but they do not demonstrate a causal link between barrier change and autonomic dysfunction [37].

In experimental hypertension and heart failure, dysfunction within the PVN, NTS, and RVLM involves both endothelial transport and junctional integrity. Angiotensin II and inflammatory signals act at the vascular interface, where they may promote glial activation and weaken autonomic control. Because interventions improve barrier and autonomic measurements together, the rodent data support an amplifying loop. Its importance in patients remains uncertain. The key experimental studies that directly assess central barrier function in cardiovascular autonomic regions are summarized in Table 1. The strongest evidence comes from models of hypertension and heart failure in which permeability or endothelial transport was assessed together with autonomic physiology, with several studies also including mechanistic or interventional approaches. Hypertension and heart failure provide stronger evidence than the other conditions reviewed because several studies measured regional BBB permeability or ultrastructure together with cardiovascular autonomic physiology in the same animals, with additional longitudinal or interventional support. By contrast, peripheral and human studies generally assessed nonautonomic structures or lacked formal autonomic testing. These mechanisms may generate hypotheses for other diseases, but they cannot be extrapolated without site-specific evidence.

Exercise and angiotensin receptor blockade also exert systemic cardiovascular and anti-inflammatory effects. Parallel improvement in barrier and autonomic measures therefore supports coupling but does not prove that restoration of barrier integrity mediated autonomic recovery. The hydralazine comparison [23] argues against blood-pressure lowering alone, whereas local IL-17 receptor A knockdown [36] supports a regional endothelial mechanism but did not include an autonomic endpoint.

**Figure 2:**
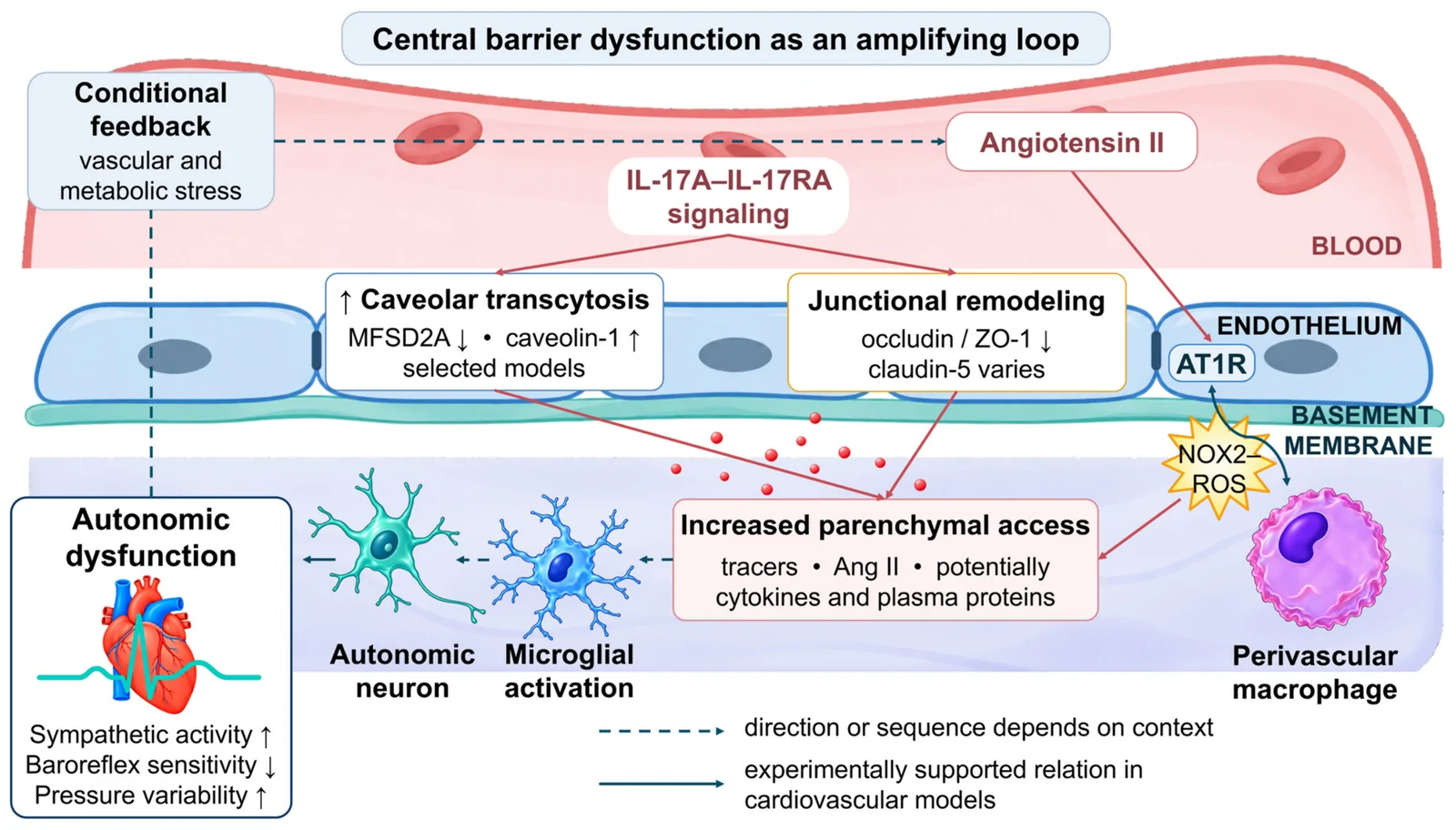
Central barrier dysfunction as an amplifying loop in cardiovascular autonomic dysregulation. Angiotensin II, IL-17A signaling, increased transcytosis, junctional remodeling, and endothelial–PVM crosstalk increase parenchymal access of circulating mediators, promoting neuroinflammation and autonomic dysfunction. Vascular and metabolic stress may reinforce this process. Solid arrows indicate experimentally supported relationships; dashed arrows indicate context dependent direction or sequence. Ang II, angiotensin II; AT1R, angiotensin II type 1 receptor; IL-17A, interleukin 17A; IL-17RA, interleukin 17 receptor A; MFSD2A, major facilitator superfamily domain containing 2A; NOX2, NADPH oxidase 2; PVM, perivascular macrophage; ROS, reactive oxygen species; ZO-1, zonula occludens 1.

Figure 2 integrates these findings into a proposed amplifying loop.

## 5. Peripheral Neural Barriers in Diabetic and Immune Autonomic Neuropathy

Diabetes is the main clinical setting in which peripheral barrier pathology could contribute to cardiovascular autonomic neuropathy (CAN). CAN disrupts neural control of the circulation, and reduced heart rate variability may precede overt cardiovascular symptoms. Low heart rate variability was used to define CAN in 4842 ACCORD participants with type 2 diabetes. During a median of 4.9 years, those meeting this definition had a higher hazard of incident silent myocardial infarction (hazard ratio 1.91, 95% confidence interval 1.14–3.20) [38]. Diabetic injury involves both the peripheral nerve and its microvasculature, with metabolic and inflammatory processes occurring together [39].

Human biopsies show that the endoneurial capillary basement membrane is thickened. Pericyte loss and endothelial hyperplasia have also been reported. Experimental evidence of altered permeability supports a contribution from the BNB, but does not establish it as the dominant cause of CAN [40]. Reduced perfusion can cause endoneurial hypoxia. Increased permeability can disturb local homeostasis and expose axons and Schwann cells to circulating factors. These processes may affect autonomic axons within mixed nerves, although most direct evidence comes from somatic structures.

A longitudinal rat study illustrates the required caution. In diabetic polyneuropathy induced with streptozotocin, the BNB and the barrier in dorsal root ganglia became permeable to small molecules after eight weeks, while larger tracers remained excluded and the blood–spinal cord barrier remained intact. Claudin-1 expression fell in the perineurium, and macrophage coverage around vessels decreased. Mechanical hypersensitivity had already appeared at two weeks, so barrier leakage followed the onset of the measured phenotype and may have maintained injury instead of initiating it [41]. The study did not measure vagal or sympathetic fibers, heart rate variability, or baroreflexes. It cannot be cited as direct proof that BNB failure causes CAN.

Human studies have examined CAN alongside generalized endothelial dysfunction and low-grade inflammation. Bhati et al. studied 50 adults with type 2 diabetes. Higher IL-6 was associated with lower total heart rate variability power (*p* = 0.009). IL-18 and high sensitivity C-reactive protein showed similar inverse associations with cardiac vagal indices, while nitric oxide measures were positively associated with vagal control [42]. A separate study compared 25 participants with impaired glucose tolerance, 25 with type 2 diabetes, and 30 controls. Von Willebrand factor and soluble E-selectin were higher in both patient groups, in which heart rate variability was also impaired—neither marker showed a strong association with heart rate variability or sympathetic skin responses [43]. The study documented vascular and autonomic abnormalities in the same participants, although their association was weak. Peripheral endothelial function does not measure permeability of the endoneurial microvasculature, and retinopathy, nephropathy, or circulating endothelial markers should not be presented as substitutes for a BNB measurement.

The current evidence supports a two-level interpretation. BNB dysfunction is established in diabetic peripheral neuropathy, and CAN often coexists with peripheral neuropathy and other microvascular complications. A systematic review of 16 observational studies (*n* = 2582) found an association between diabetic peripheral neuropathy and CAN, with more severe peripheral neuropathy accompanying worse autonomic-test results, but the included studies did not identify a barrier lesion as the shared cause [44]. A direct chain from a measured BNB lesion in an autonomic nerve to a quantified cardiovascular autonomic deficit has not been demonstrated in humans or in a dedicated animal experiment. Studies of the vagus nerve, sympathetic chain, cardiac autonomic branches, and stellate ganglion are needed before BNB failure can be called a pivotal event in CAN.

Inflammatory neuropathies provide clearer evidence that the BNB opens, but the autonomic inference is again indirect. Guillain–Barré syndrome can cause severe autonomic dysfunction, including cardiac arrhythmias and blood-pressure instability [45]. In experimental autoimmune neuritis in rats, Evans blue–albumin (approximately 69 kDa) crossed sciatic-nerve endoneurial vessels from disease onset, and circulating nanoparticles of 50–150 nm accumulated according to disease stage and particle size. CD68-positive macrophages increased from 7.9 ± 4.0 cells/mm^2^ in naïve nerves to 678 ± 94.5 cells/mm^2^ at peak disease (*n* = 3–4 rats per stage) [46]. In a coculture of human peripheral nerve microvascular endothelial cells and pericytes, immunoglobulin G from patients with typical or multifocal chronic inflammatory demyelinating polyneuropathy increased 10 kDa dextran permeability, while immunoglobulin G from patients with multifocal motor neuropathy increased TNF-α and VCAM-1 without a reported permeability increase [47]. These findings show feasible routes for immune access in inflammatory neuropathies, but they concern sciatic nerve or an in vitro barrier and do not establish cardiovascular autonomic causation in Guillain–Barré syndrome.

Autoimmune autonomic ganglionopathy presents a different problem. Whole-cell patch clamp in cultured human IMR-32 cells showed that immunoglobulin G from seven affected patients progressively reduced currents through ganglionic nicotinic acetylcholine receptors, while immunization against the α3 receptor subunit produced autonomic deficits in mice [48,49]. These studies establish pathogenic action at the receptor. They do not demonstrate a preceding lesion of a ganglionic vascular barrier. Given the normal heterogeneity of vascular permeability across autonomic ganglia, antibody access may depend on ganglion and vascular territory and may not require acquired barrier failure [19,20]. The original claim that antibodies must first cross an endothelial barrier is consequently too strong.

Peripheral data thus complement, but do not mirror, the central literature. In autonomic nuclei, experiments directly combine leakage with sympathetic or baroreflex measurements. Peripheral studies usually relate leakage to nonautonomic outcomes, so its contribution to autonomic dysfunction remains inferred. This distinction limits the therapeutic conclusions that can be drawn.

## 6. Mechanisms That Can Connect Barrier Failure with Autonomic Dysfunction

The first mechanism is altered endothelial transport. Transcytosis permits macromolecules to cross an endothelial cell without visible separation of tight junctions. MFSD2A normally suppresses caveola formation in brain endothelium, and its loss increases caveolar transport [11]. Pericytes also restrain vesicular traffic and preserve endothelial polarity [10]. In aged mice, plasma protein transport shifts from pathways mediated by receptors toward caveolar transcytosis as pericyte coverage falls, showing that transport remodeling cannot be reduced to unrestricted leakage [50]. The hypertension and heart failure studies show increased caveolin-1 and endothelial vesicles in autonomic nuclei, sometimes in the absence of an early decrease in claudin-5 [28,34]. A selective change in transcytosis can thus precede broad paracellular failure.

Direct ultrastructural evidence of increased endothelial vesicle number or trafficking was provided in the heart failure studies [34,35]. In the hypertension and experiments related to IL-17A, a caveolar route was inferred mainly from tracer leakage together with changes in caveolin-1 or MFSD2A [28,33,36]; expression of these proteins alone does not quantify transcytotic flux.

Junctional remodeling offers another route to barrier failure. Paracellular permeability depends strongly on claudin-5, together with the wider junctional complex and its linkage to the cytoskeleton. In adult mice, even mosaic deletion of claudin-5 causes rapid leakage and glial activation despite residual expression in many vessels [12]. Occludin deficiency also worsens tracer leakage after experimental stroke [51]. In cardiovascular models, the pattern varies: claudin-5 is preserved in some stages of hypertension, proteins associated with tight junctions fall in the heart failure model involving IL-17A, and both transcellular and paracellular routes change after coronary ligation [28,34,36]. A recent review summarizes the molecular regulation and dysfunction of endothelial tight junctions related to the disease [52]. Paracellular involvement was supported by shorter junctional contacts on electron microscopy in heart failure [34,35] and by changes in claudin-5, occludin, or ZO-1 [34,36]. Changes in junctional protein abundance alone, however, do not directly measure paracellular permeability.

The third mechanism is a vascular and immune feedback loop. Angiotensin II stimulates NOX2 at the neurovascular interface, raising oxidative stress and weakening endothelial control of transport. Greater access of circulating peptides and cytokines to the parenchyma may prolong microglial and neuronal activation [23,24,25]. In PVN vessels, IL-17A may add to this effect by increasing caveolar transport and weakening junctions [33,36].

Other models illustrate individual parts of the proposed mechanism. In chronic cerebral hypoperfusion, endothelial VCAM1 contributed to tracer leakage, although the affected white matter was not an autonomic region [53]. Cadmium exposure activated microglia in the RVLM and disrupted cardiovascular regulation without a barrier measurement. Without a permeability measurement, the cadmium findings provide evidence of RVLM neuroinflammation only [54]. In another model, extracellular vesicles from mouse neutrophils exposed to LPS increased cerebral leakage after systemic administration. They also increased permeability in cultured brain endothelial monolayers [55]. That work did not examine autonomic nuclei or autonomic physiology. The relative order of these events differs between models and should not be reduced to one universal sequence.

Plasma proteins provide another possible effector. Fibrinogen that enters the brain can bind the CD11b/CD18 integrin on microglia, activate NOX2, and promote elimination of dendritic spines. Blocking this interaction or reactive oxygen species prevents spine loss in an Alzheimer disease model [56]. Human and mouse traumatic brain injury data extend the fibrin–CD11b mechanism to innate immune activation and oxidative injury [57]. Complement signaling also enables microglia to remove synapses in experimental neurodegeneration [58]. These studies establish mechanisms outside cardiovascular autonomic circuits; they did not examine the PVN, NTS, or RVLM. Fibrinogen leakage in an autonomic nucleus could use the same pathway, but this remains an inference until synapses and autonomic output are measured in the same experiment.

A change in the extracellular environment may affect autonomic neurons even without structural synapse loss. Entry of albumin and ions alters osmotic balance and buffering. Cytokines change neuronal membrane conductance and glial uptake of glutamate and potassium. Edema can increase diffusion distances and reduce capillary exchange. In the PVN and RVLM, even modest changes in the balance of excitation and inhibition can alter sympathetic output. In the NTS, they may impair the transmission of baroreceptor input. This mechanism is physiologically plausible but is rarely examined together with permeability and neuronal activity in the same animals.

Peripheral nerves add ischemia and endoneurial pressure to the model. The endoneurial space has limited capacity to accommodate edema. Reduced perfusion or impaired autoregulation can compromise oxygen delivery, while basement membrane thickening increases the diffusion distance. If permeability also rises, Schwann cells and axons are exposed to both metabolic and inflammatory stress [40]. Long, distal axons are particularly vulnerable, but the common description of a barrier lesion causing progressive distal axon loss exceeds the direct evidence. Axonal degeneration and microvascular pathology coexist, but their sequence differs between diseases and stages. Inflammation or angiotensin II may first alter endothelial transport and initiate leakage. Circulating factors that enter neural tissue can then affect glia and neurons, changing autonomic output. The resulting cardiovascular or metabolic strain may further damage the vascular interface.

A useful model must allow the initiating event to differ among diseases. It must also allow barrier dysfunction to be secondary in some settings. This conditional model is better supported than a claim that BBB or BNB failure is a shared primary cause of all autonomic neurodegeneration. The evidence reviewed above indicates that neural barrier dysfunction does not represent a uniform pathological state. Instead, the predominant barrier phenotype, its underlying mechanisms, and its potential autonomic consequences vary according to anatomical site and disease context, as summarized in Table 2. Taken together, the literature supports three nonexclusive roles for barrier dysfunction: an upstream lesion in selected experimental settings, a consequence of hypertension, inflammation, or neural injury, and a secondary amplifier. The amplifying role has the strongest support in central rodent models, whereas an initiating role in human dysautonomia remains unproven.

## 7. Human Evidence, Biomarkers, and Therapeutic Implications

Human evidence is strongest for the presence of barrier abnormalities, not for their autonomic consequences. The heart failure study using water exchange imaging demonstrates altered BBB physiology in living patients but does not pair the regional findings with formal autonomic reflex tests [37]. MRI with dynamic contrast enhancement showed higher Ktrans in 49 patients with Parkinson’s disease than in 31 controls. The largest differences occurred in the substantia nigra, with additional changes in posterior cortical regions and white matter [59]. A separate study used human Transwell cultures and BBB chips generated from induced pluripotent stem cells. Erythrocyte extracellular vesicles obtained from participants with Parkinson’s disease crossed the endothelial layer by transcytosis involving caveolin. Junctional protein expression subsequently declined, and dopaminergic neurons were injured [60]. These in vitro models did not test autonomic pathways. Parkinson’s disease often includes autonomic dysfunction, yet neither study showed that permeability explains the autonomic phenotype.

Animal and human studies are not directly comparable. Rodent experiments used regional vascular tracers, immunostaining, and electron microscopy, often together with baroreflex sensitivity, cardiovascular spectral indices, or sympathetic measures. Human studies used water-exchange arterial spin labeling, dynamic contrast-enhanced MRI, postmortem extravascular proteins, Qalb, or circulating markers, and most lacked a prespecified autonomic outcome. These methods assess different transport processes, molecular sizes, spatial scales, and disease stages.

Multiple system atrophy provides pathological evidence in a disorder defined by severe autonomic failure. Examination of postmortem tissue from patients with multiple system atrophy revealed lower endothelial claudin-5 and leakage of fibrinogen and immunoglobulin G. Phosphorylated α-synuclein was present in nearby oligodendrocyte precursor cells [61]. Another postmortem study reported vascular complement deposition, extravascular fibrinogen, and C1q-positive microglia around cerebral vessels [62].

PET provided evidence of glial inflammation but did not measure permeability. One study used [11C](R)-PK11195 in 14 patients with the parkinsonian form of multiple system atrophy. A later multicenter study used [11C]PBR28 in 66 patients with multiple system atrophy and 24 with Parkinson’s disease [63,64]. These findings support the presence of vascular injury and inflammation, but do not establish whether BBB leakage precedes or contributes to degeneration of medullary autonomic neurons. Parkinson’s disease and multiple system atrophy are therefore included as human comparison disorders in which barrier abnormalities coexist with dysautonomia or autonomic failure. They support biological plausibility but not the central causal hypothesis, because the reported barrier changes were not localized to cardiovascular autonomic circuits and their temporal relation to neurodegeneration is unknown.

The cerebrospinal fluid to serum albumin quotient, Qalb, is often described too broadly. It quantifies the relation between albumin concentrations in cerebrospinal fluid and serum and is influenced by transfer across the blood–cerebrospinal fluid interfaces, cerebrospinal fluid flow, age, and systemic albumin. It does not identify the anatomical site of leakage and is not a regional measure of BBB permeability. In 68 patients with early Parkinson’s disease, higher Qalb was associated with lower alpha-band cortical connectivity, but Qalb was not associated with the clinical features examined [65]. This result is relevant to brain network function, not proof that Qalb tracks dysautonomia.

Imaging measurements also require precise interpretation. Ktrans from dynamic contrast-enhanced MRI reflects contrast transfer under a kinetic model and can be influenced by perfusion, vascular surface area, acquisition, and model choice. Water exchange from arterial spin labeling examines a different transport property. Neither value should be described simply as BBB breakdown. A recent review identifies variability in protocols, tracers, input functions, and parameter estimation as major limits on cross-study comparison [66]. Regional imaging of the PVN, NTS, and RVLM is technically difficult because these structures are small and close to cerebrospinal fluid spaces and large vessels. Reproducible acquisition and anatomical segmentation are prerequisites for testing the cardiovascular autonomic hypothesis in patients.

Circulating claudin-5, occludin, ZO-1, soluble adhesion molecules, and extracellular vesicles are potential markers of endothelial injury, but none is currently a validated marker of neural barrier dysfunction in dysautonomia. Reviews of BBB biomarkers distinguish structural, circulating, and imaging measures and caution against treating them as interchangeable [67]. Plasma junctional proteins have been measured after intracranial hemorrhage, which supports analytical feasibility but not anatomical specificity [68]. In cultured BBB models, hyperglycemia and advanced glycation end products promote release of occludin and claudin-5 on extracellular microvesicles [69]. This observation offers a mechanism for a circulating signal, not evidence that the signal localizes a barrier lesion in a patient. Cerebrospinal fluid soluble PDGFRβ has also been studied as a marker linked to pericytes across neurodegenerative disorders, but it reports one component of NVU injury and cannot by itself quantify regional permeability [70]. Because these proteins are not confined to neural vessels, their circulating concentrations cannot by themselves identify the source or mechanism of barrier injury. A biomarker panel would need to demonstrate a reproducible relation with an imaging or cerebrospinal fluid measure and with a validated autonomic endpoint. In this review, von Willebrand factor, soluble E-selectin, cytokines, and circulating junctional proteins are treated as markers of general endothelial injury or inflammation. Regional imaging, tracer flux, and extravascular plasma proteins provide more proximal evidence of a neural interface abnormality, although each remains limited by anatomical and mechanistic specificity; Qalb primarily reflects blood–cerebrospinal fluid exchange and cerebrospinal fluid dynamics.

Heart rate variability, baroreflex sensitivity, catecholamine concentrations, microneurography, and autonomic reflex tests measure different aspects of autonomic function. They are outcomes of interest, not surrogate markers of the NVU. Heart rate variability is affected by respiration, rhythm, medication, age, activity, and recording duration. Recent reviews support heart rate variability as a measure of neurocardiac function and as a candidate marker of aging or inflammation, not as a direct measure of the NVU [71,72]. Improvement in heart rate variability after exercise cannot by itself demonstrate barrier repair. The rodent heart failure studies are informative because permeability and autonomic measurements were made in the same experimental design [34,35]. Human studies should adopt the same principle with methods appropriate for patients. Across the reviewed studies, total power, LF/HF, and vagal indices were obtained under different recording durations and experimental conditions. These measures are not directly interchangeable and were therefore interpreted within individual studies rather than compared quantitatively.

The experimental treatment data identify pathways, not clinical indications. Exercise training improves barrier measurements and autonomic control in rat hypertension and heart failure [28,29,34]. Losartan produces similar effects in the relevant models [23,35]. TLR4 inhibition and modulation of the RORγt–IL-17A axis also reduce leakage and sympathetic activation [25,33]. Exercise and angiotensin receptor blockers already have established cardiovascular indications, but their clinical benefit cannot be attributed to neural barrier repair without direct human evidence. Systemic inhibition of TLR4 or IL-17 pathways carries immune consequences and has not been validated for this purpose.

A barrier can also hinder therapy. Temporary opening may improve drug delivery, while chronic nonspecific opening may expose neural tissue to harmful proteins and immune mediators. A phase I study in five patients with Parkinson’s disease dementia demonstrated that focused ultrasound opening could be focal and reversible, but the study addressed feasibility and did not establish clinical efficacy or relevance to autonomic failure [73]. Treatment should consequently aim to normalize the abnormal route of transport, not to make every neural barrier maximally restrictive. In a state dominated by excessive caveolar transport, restoration of MFSD2A signaling or control of caveola formation might be rational. In a state dominated by leukocyte adhesion or junctional loss, another strategy would be required. No such approach has yet shown clinical efficacy for autonomic failure.

Peripheral and human evidence relevant to neural barrier involvement in autonomic disorders is summarized in Table 3. In contrast to the central experimental literature, most studies do not combine direct assessment of a neural barrier with cardiovascular autonomic measurements, and the link to autonomic dysfunction is often indirect.

## 8. Evidence Gaps and Future Perspectives

The central field needs replication outside the spontaneously hypertensive rat and coronary ligation models [23,25,35]. Experiments should include both sexes, several ages, clinically relevant comorbidities, and interventions that separate blood pressure lowering from direct action on the vascular interface. Regional tracer studies should use molecules of more than one size and combine them with electron microscopy or quantitative measures of vesicular transport and junctional continuity. Permeability, neuronal activity, and autonomic physiology should be measured at matched time points.

The peripheral field needs anatomical sampling that is actually autonomic. Results from sciatic or sural nerves and dorsal root ganglia cannot be assumed to represent autonomic structures [8,22,40,41]. Direct work is needed in the vagal and sympathetic pathways that supply the cardiovascular system. In diabetes models, barrier measurements at these sites should be aligned with cardiovascular reflex testing and telemetry, then related to structural changes in autonomic fibers. This design would determine whether leakage precedes CAN, follows it, or marks a parallel consequence of systemic microvascular disease.

Autonomic ganglia require a vascular atlas across species and anatomical levels. Endothelial fenestration, junctional proteins, transporter expression, tracer permeability, macrophage coverage, and proximity to principal neurons should be mapped in the same tissue. Without that baseline, pathological permeability cannot be distinguished from normal access of circulating molecules [19,20]. The issue is especially important for autoimmune autonomic ganglionopathy, where antibody pathogenicity is established [48,49], but vascular access remains poorly defined.

Human studies should combine a regional barrier method with a prespecified autonomic phenotype. In heart failure and hypertension, this could include permeability or water exchange imaging [37] with muscle sympathetic nerve activity, baroreflex sensitivity, standardized heart rate variability, and medication data. In diabetes, corneal confocal microscopy can detect small fiber loss in patients with diabetic autonomic neuropathy [74], and skin biopsy can provide a complementary structural measure. Neither method directly measures the peripheral neural microvasculature, so a direct barrier method or a validated vascular proxy is still needed. Longitudinal sampling before and after an intervention is more informative than a cross-sectional correlation.

Biomarker development should proceed through validation against anatomy. Qalb, circulating junctional proteins, soluble adhesion molecules, extracellular vesicles, and TSPO positron emission tomography report different biological processes [43,64,65,68,69]. A composite panel may be useful, but only if each component has a defined role and the panel predicts a barrier measurement or future autonomic decline beyond conventional risk factors. Treating several nonspecific markers as interchangeable evidence of NVU injury would reproduce the central weakness of the original broad narrative.

Finally, causal language should follow the experiment. Demonstration of leakage and autonomic dysfunction in the same disease supports coexistence. A temporal sequence supports direction. An intervention that repairs the barrier and restores function supports coupling. Endothelial manipulation that changes autonomic output while other major pathways remain controlled provides stronger causal evidence. This hierarchy should guide both future work and interpretation of the current literature. Future studies should be designed explicitly to discriminate among initiating, consequential, and amplifying roles rather than treating any permeability change as causal.

## 9. Conclusions

Changes in neural barriers may contribute to cardiovascular autonomic dysfunction, but the strength of the evidence differs between parts of the nervous system. The clearest findings come from experimental studies of hypertension and heart failure. Changes in BBB permeability were observed in central autonomic nuclei, and several interventions improved both barrier function and autonomic measurements. These results support a connection between the BBB and autonomic control, although they do not establish that BBB damage initiates the disease.

Evidence outside central autonomic nuclei is much less complete. Studies of diabetes and inflammatory neuritis show changes in the BNB, but mainly in sensory or somatic nerves. The vasculature of autonomic ganglia remains poorly characterized and cannot be described using a single barrier model. Human studies have detected barrier abnormalities in heart failure and synucleinopathies, but barrier function and autonomic physiology have rarely been assessed in the same patients.

Current evidence does not support BBB or BNB dysfunction as a common initiating cause of dysautonomia. It most strongly supports a secondary amplifying role in experimental hypertension and heart failure. In peripheral and neurodegenerative disease, barrier changes may instead be consequences or parallel manifestations, and an initiating role in human cardiovascular dysautonomia remains unproven. Future studies should examine defined autonomic structures, establish temporal order, and measure barrier function together with physiological autonomic outcomes.

## Figures and Tables

**Table 1:** Key experimental evidence linking central neural barrier dysfunction with cardiovascular autonomic abnormalities.

Study, Model, Region and Sample Size	Barrier Method and Findings	Autonomic Findings	Evidence Type and Mechanistic or Intervention Findings
Spontaneously hypertensive rats; PVN, NTS, RVLM; BBB *n* = 8/group; treated SHR *n* = 4/group [23]	Assessed with intracarotid 10 kDa FITC-dextran and 70 kDa rhodamine-dextran and fluorescence imaging. Increased tracer permeability; circulating angiotensin II detected outside vessels near neurons and microglia.	Central autonomic regions were studied in hypertension; the experiment primarily established access of circulating angiotensin II to autonomic nuclei rather than a full autonomic test battery.	Indirect evidence. Losartan prevented BBB abnormalities, whereas hydralazine lowered arterial pressure without equivalent BBB protection, supporting angiotensin II type 1 receptor signaling beyond pressure alone.
Spontaneously hypertensive rats; PVN, NTS, RVLM; BBB *n* = 4/subgroup; autonomic *n* = 4–10/subgroup [29]	Assessed by fluorescence imaging after intracarotid administration of 10 kDa FITC-dextran and 70 kDa rhodamine-dextran. Marked 10 kDa FITC-dextran leakage was observed in established hypertension and was reduced early during exercise training.	Leakage reduction occurred together with lower cardiac LF/HF ratio and reduced vasomotor sympathetic activity, before the later fall in resting pressure.	Direct interventional evidence. Exercise training reduced local angiotensin II availability and microglial activation; BBB improvement correlated with autonomic improvement.
Angiotensin II-induced hypertension in mice; cerebral arterioles and venules; primary BBB comparison *n* = 13–14/group [24]	Assessed with 3–4 kDa FITC-dextran, in vivo two-photon microscopy, and electron microscopy. Angiotensin II increased BBB permeability, particularly in arterioles and venules larger than 10 µm.	No formal autonomic reflex endpoint was reported; the study provides mechanistic BBB evidence in a hypertension model.	Indirect evidence. Endothelial angiotensin II type 1 receptors initiated BBB disruption; perivascular macrophage angiotensin II type 1 receptor–NOX2 signaling amplified the response.
Spontaneously hypertensive rats; PVN, NTS, RVLM; BBB *n* = *6*/group; autonomic *n* = 5–10/group [25]	Assessed with 10 kDa FITC-dextran and 70 kDa rhodamine-dextran and confocal imaging. Increased BBB permeability accompanied by microglial activation and increased IL-6 and TNF-α expression.	Impaired baroreflex sensitivity and increased sympathetic contribution to arterial pressure.	Direct interventional evidence for TLR4 blockade. TLR4 inhibition improved BBB integrity, reduced neuroinflammation, and improved autonomic measures. AT1R inhibition improved BBB and neuroinflammatory measures but was not assessed in the autonomic subcohort.
Spontaneously hypertensive rats during transition from prehypertension to hypertension; PVN, NTS, RVLM; *n* = 3 (BBB) and *n* = 6–9 (autonomic) per group at each age [27]	Assessed with 10 kDa FITC-dextran and 70 kDa rhodamine-dextran and fluorescence imaging. Local angiotensin II signaling increased before clear tracer leakage; permeability and microglial activation increased as hypertension developed.	Barrier changes emerged as arterial pressure and autonomic abnormalities developed.	Direct temporal evidence across age-specific cohorts. The temporal sequence supports coupling but does not establish that BBB leakage initiates the first rise in arterial pressure.
Spontaneously hypertensive rats; PVN; *n* = 7–8/group [28]	Assessed with 10 kDa FITC-dextran and 70 kDa rhodamine-dextran and caveolin-1 and claudin-5 expression analyses. Tracer leakage and caveolin-1 increased; claudin-5 was not reduced in parallel.	Autonomic indices improved during exercise training.	Direct interventional evidence. Exercise reduced caveolin-1 expression and tracer leakage and improved autonomic control. Transcytosis was inferred rather than directly quantified.
Coronary ligation-induced heart failure in rats; PVN, NTS, RVLM; autonomic *n* = 6–7/group; BBB/TEM *n* = 3/group [34]	Assessed with 10 kDa FITC-dextran and 70 kDa rhodamine-dextran and transmission electron microscopy. Increased BBB permeability; more endothelial vesicles, shorter junctional contacts, increased caveolin-1, and reduced claudin-5.	Increased vasomotor sympathetic activity and arterial pressure variability, with reduced cardiac parasympathetic control and impaired reflex sensitivity.	Direct interventional evidence. Exercise improved permeability, ultrastructural barrier features, and autonomic control despite persistent cardiac injury.
Myocardial infarction-induced heart failure in rats; PVN; BBB and molecular analyses *n* = 6/group [36]	Assessed with 10 kDa FITC-dextran and 70 kDa rhodamine-dextran and confocal imaging. Tracer leakage increased in the PVN but not in the sampled cortex; caveolin-1 increased, whereas occludin and ZO-1 decreased.	No direct cardiovascular autonomic endpoint was reported in this experiment.	Indirect evidence. Plasma IL-17A correlated with PVN leakage; local IL-17 receptor A knockdown reduced permeability, lowered caveolin-1, and restored occludin and ZO-1.
Coronary ligation-induced heart failure in rats; PVN; autonomic *n* = 6/group; BBB/TEM *n* = 3/group [35]	Assessed with 10 kDa FITC-dextran and 70 kDa rhodamine-dextran and transmission electron microscopy. Increased permeability, vesicular trafficking, and tight junction weakness.	High sympathetic activity and pressure variability with reduced cardiac parasympathetic control.	Direct interventional evidence. Exercise and losartan similarly reduced angiotensin II, leakage, vesicular transport, junctional weakness, microglial activation, and autonomic imbalance; combined treatment showed no clear additional benefit.
Angiotensin II-induced hypertension in rats; PVN; autonomic *n* = 5–6/group; BBB and molecular analyses *n* = 6/group [33]	Assessed with 10 kDa FITC-dextran and 70 kDa rhodamine-dextran and fluorescence imaging. Reduced MFSD2A, increased caveolin-1, and increased tracer leakage.	Increased sympathetic tone and arterial pressure.	Direct interventional evidence. RORγt inhibition preserved MFSD2A, reduced caveolin-1 and leakage, limited microglial activation, and reduced sympathetic tone and arterial pressure.

Note: Sample sizes refer to the assays relevant to the summarized findings and do not necessarily represent the total number of animals enrolled. Direct evidence denotes assessment of both barrier and cardiovascular autonomic outcomes within the same experimental study and model. Indirect evidence denotes barrier assessment without a formal cardiovascular autonomic endpoint. Neither classification alone establishes causal mediation. Abbreviations: BBB, blood–brain barrier; FITC, fluorescein isothiocyanate; IL-6, interleukin 6; IL-17A, interleukin 17A; LF/HF, low frequency to high frequency ratio; MFSD2A, major facilitator superfamily domain containing 2A; NOX2, NADPH oxidase 2; NTS, nucleus tractus solitarius; PVN, paraventricular nucleus of the hypothalamus; RORγt, retinoic acid receptor related orphan receptor γt; RVLM, rostral ventrolateral medulla; TNF-α, tumor necrosis factor alpha; ZO-1, zonula occludens 1.

**Table 2:** Barrier phenotypes and their proposed relevance to cardiovascular autonomic dysfunction.

Neural Interface and Setting	Predominant Barrier Phenotype	Candidate Mechanism	Proposed Autonomic Implication
Central BBB in hypertension: PVN, NTS, and RVLM	Regional increase in permeability. In the PVN, increased endothelial transcytosis can occur without a parallel reduction in claudin-5 early in the process [23,27,28]	Angiotensin II signaling through the angiotensin II type 1 receptor, increased caveolar transport, Toll-like receptor 4 signaling, and interactions between endothelium, perivascular macrophages, and microglia [24,25]	Increased access of circulating signals to autonomic nuclei may amplify sympathoexcitation and impaired baroreflex control. Exercise or receptor blockade improves barrier and autonomic measures within the same experimental setting [25,29]
Central BBB in heart failure: PVN, NTS, and RVLM	Increased permeability accompanied by greater endothelial vesicular transport and junctional abnormalities. In the PVN, caveolin-1 increases and junctional proteins may decline [34,35,36]	Angiotensin II and interleukin 17A signaling can promote caveolar transport and junctional remodeling [35,36]	Barrier abnormalities occur together with increased sympathetic activity and reduced cardiac parasympathetic and reflex control. Exercise and losartan improve both domains [34,35]
Endoneurial BNB in diabetes	Endoneurial microvascular remodeling, altered perfusion, edema, and selective permeability according to molecular size; reduced perineurial claudin-1 has been demonstrated experimentally [40,41]	Altered perfusion and permeability may disturb endoneurial fluid and ionic homeostasis, reduce oxygen delivery, and increase exposure to circulating metabolic and inflammatory factors [40]	These changes could affect autonomic axons travelling within mixed nerves. Diabetic peripheral neuropathy is associated with cardiovascular autonomic neuropathy, but a direct link between a measured BNB lesion in an autonomic nerve and cardiovascular autonomic dysfunction has not been demonstrated [44]
BNB in inflammatory neuropathy	Endoneurial permeability increases during experimental autoimmune neuritis, allowing entry of albumin and nanoparticles. Patient immunoglobulin G can also increase permeability in a human BNB coculture model [46,47]	Increased vascular permeability and endothelial activation may facilitate access of circulating immune mediators to peripheral nerve tissue [46,47]	This provides a plausible route for injury to autonomic fibers in disorders such as Guillain–Barré syndrome, but the direct barrier evidence comes from sciatic nerve or in vitro models, not cardiovascular autonomic pathways [45,46,47]
Autonomic ganglion vasculature	Baseline permeability is heterogeneous between sympathetic ganglia and even between vascular territories within the same ganglion; both restrictive and more permissive vascular phenotypes occur [19,20]	Access of circulating molecules may depend on ganglion, vascular territory, tracer size, and endothelial phenotype and does not necessarily require acquired barrier failure [19,20]	Ganglionic nicotinic acetylcholine receptor antibodies can directly impair ganglionic transmission and produce autonomic dysfunction, but the vascular route by which antibodies reach their targets remains unresolved [48,49]

Abbreviations: BBB, blood–brain barrier; BNB, blood–nerve barrier; NTS, nucleus tractus solitarius; PVN, paraventricular nucleus of the hypothalamus; RVLM, rostral ventrolateral medulla.

**Table 3:** Peripheral and human evidence relevant to neural barrier dysfunction in dysautonomia.

Study, Sample Size (When Available), Species/Model, and Neural Interface	Barrier or Vascular Method and Findings	Autonomic Outcome	Directness of Evidence and Main Limitation
Diabetic polyneuropathy in rats induced with streptozotocin; BNB and dorsal root ganglion barrier [41]	After eight weeks, small-molecule permeability increased while larger tracers remained excluded; claudin-1 decreased in the perineurium and vessel-associated macrophage coverage fell.	Diabetes is directly relevant to cardiovascular autonomic neuropathy.	Indirect evidence from sensory structures. Mechanical hypersensitivity preceded barrier leakage, and cardiovascular autonomic pathways or outcomes were not assessed.
50 adults with type 2 diabetes; systemic endothelial and inflammatory markers [42]	IL-6, IL-18, high-sensitivity C-reactive protein, nitric oxide, and endothelial nitric oxide synthase were assessed together with autonomic measures.	Higher inflammatory markers were associated with lower heart rate variability measures; nitric oxide measures showed positive associations with vagal indices.	Indirect and nonspecific evidence. Circulating inflammatory and endothelial markers were associated with autonomic measures, but no BBB or BNB measurement was performed.
25 impaired-glucose-tolerance participants, 25 with type 2 diabetes, 30 controls; systemic endothelium [43]	von Willebrand factor and soluble E-selectin were higher in patient groups.	Heart rate variability was impaired; sympathetic skin response was also assessed.	Indirect and nonspecific evidence. Systemic endothelial markers were weakly related to autonomic measures and did not assess the BNB.
Systematic review of 16 observational studies, *n* = 2582; diabetic peripheral neuropathy and cardiovascular autonomic neuropathy [44]	No direct neural barrier measurement; review addressed coexistence of peripheral and cardiac autonomic neuropathy.	Diabetic peripheral neuropathy was associated with cardiovascular autonomic neuropathy, with more severe peripheral neuropathy accompanying worse autonomic-test results.	Indirect evidence. Coexistence of peripheral and cardiovascular autonomic neuropathy did not identify a shared barrier lesion.
Experimental autoimmune neuritis in rats; sciatic nerve BNB; assay-specific *n* = 3–5 rats/group [46]	Evans blue–albumin and 50–150 nm nanoparticles entered the endoneurial compartment, with permeability depending on disease stage and nanoparticle size; macrophage accumulation increased markedly.	Provides a feasible route for immune access in inflammatory neuropathy, a disease class that can include autonomic dysfunction.	Indirect evidence from a somatic nerve model. Autonomic nerves and cardiovascular autonomic outcomes were not assessed.
Human peripheral nerve microvascular endothelial cell–pericyte coculture; inflammatory neuropathies, typical CIDP *n* = 15; multifocal CIDP *n* = 14; MMN *n* = 12; healthy controls *n* = 14 [47]	Immunoglobulin G from typical or multifocal chronic inflammatory demyelinating polyneuropathy increased 10 kDa dextran permeability; multifocal motor neuropathy immunoglobulin G increased TNF-α and VCAM-1 without a reported permeability increase.	Demonstrates that patient immunoglobulin G can alter a human BNB model.	Indirect mechanistic evidence from an in vitro somatic BNB model. Cardiovascular autonomic pathways were not assessed.
Autoimmune autonomic ganglionopathy; ganglionic nicotinic acetylcholine receptor; patient IgG *n* = 7; immunization model *n* = 27 female C57BL/6 mice (*n* = 9/group) [48,49]	No direct vascular barrier measurement.	Patient immunoglobulin G reduced ganglionic nicotinic acetylcholine receptor currents; α3-subunit immunization produced autonomic deficits in mice.	Direct evidence of receptor pathogenicity but indirect evidence of vascular access. No barrier measurement was performed.
27 adults with heart failure and 59 controls; BBB water exchange [37]	Arterial spin labeling with diffusion weighting showed altered BBB water exchange and arterial transit time in several cortical and subcortical regions.	Heart failure commonly includes autonomic dysfunction, and some affected regions participate in autonomic regulation.	Indirect human evidence. No formal autonomic reflex testing was performed, and water exchange is not equivalent to macromolecular permeability.
49 patients with Parkinson’s disease and 31 controls; BBB [59]	Dynamic MRI with contrast enhancement showed higher Ktrans, particularly in the substantia nigra, posterior cortical regions, and white matter.	Parkinson’s disease commonly includes autonomic dysfunction.	Indirect human evidence. Regional permeability was not linked to a cardiovascular autonomic outcome.
Human postmortem multiple system atrophy; cerebral microvasculature [61,62]	Reduced endothelial claudin-5 and extravasation of fibrinogen and immunoglobulin G; vascular complement deposition, fibrinogen leakage, and C1q-positive microglia were also reported.	Multiple system atrophy is characterized by severe autonomic failure.	Indirect postmortem evidence. The cross-sectional findings do not establish whether BBB leakage preceded or contributed to degeneration of medullary autonomic neurons.

Abbreviations: BBB, blood–brain barrier; BNB, blood–nerve barrier; IL-6, interleukin 6; IL-18, interleukin 18; Ktrans, volume transfer constant; TNF-α, tumor necrosis factor alpha; VCAM-1, vascular cell adhesion molecule 1.

## Data Availability

No new data were created or analyzed in this study. Data sharing is not applicable to this article.

## Abbreviations

The following abbreviations are used in this manuscript:

ACCORD	Action to Control Cardiovascular Risk in Diabetes
AT1	angiotensin II type 1 receptor
B1	kinin B1 receptor
BBB	blood–brain barrier
BNB	blood–nerve barrier
C1q	complement component 1q
CAN	cardiovascular autonomic neuropathy
CD11b	cluster of differentiation 11b
CD18	cluster of differentiation 18
CD163	cluster of differentiation 163
CD68	cluster of differentiation 68
FITC	fluorescein isothiocyanate
HMGB1	high mobility group box 1
IL-6	interleukin 6
IL-17	interleukin 17
IL-17A	interleukin 17A
IL-18	interleukin 18
Ktrans	volume transfer constant
LF/HF	low frequency to high frequency ratio
LPS	lipopolysaccharide
MFSD2A	major facilitator superfamily domain containing 2A
MRI	magnetic resonance imaging
NOX2	NADPH oxidase 2
NTS	nucleus tractus solitarius
NVU	neurovascular unit
PDGFRβ	platelet derived growth factor receptor beta
PET	positron emission tomography
PVN	paraventricular nucleus of the hypothalamus
Qalb	cerebrospinal fluid to serum albumin quotient
RNA	ribonucleic acid
RORγt	retinoic acid receptor related orphan receptor γt
RVLM	rostral ventrolateral medulla
TLR4	Toll-like receptor 4
TNF-α	tumor necrosis factor alpha
TSPO	translocator protein
VCAM-1	vascular cell adhesion molecule 1
ZO-1	zonula occludens 1

## Appendix A

The final PubMed search was conducted using the following query:

(((“neurovascular unit”[tiab] OR “neurovascular barrier”[tiab] OR “neurovascular barriers”[tiab] OR “blood-brain barrier”[tiab] OR “blood brain barrier”[tiab]) AND (hypertension[tiab] OR hypertensive[tiab] OR “heart failure”[tiab] OR “myocardial infarction”[tiab] OR autonom*[tiab] OR sympath*[tiab] OR baroreflex*[tiab] OR “paraventricular nucleus”[tiab] OR “nucleus tractus solitarius”[tiab] OR “nucleus of the solitary tract”[tiab] OR “rostral ventrolateral medulla”[tiab] OR “angiotensin II”[tiab] OR exercise[tiab]) AND (dysfunct*[tiab] OR breakdown[tiab] OR leak*[tiab] OR impair*[tiab] OR permeab*[tiab] OR transcyt*[tiab] OR “tight junction”[tiab] OR “tight junctions”[tiab] OR junction*[tiab] OR claudin*[tiab] OR occludin[tiab] OR endotheli*[tiab] OR macrophage*[tiab] OR microglia*[tiab] OR neuroinflamm*[tiab] OR inflamm*[tiab] OR integrity[tiab] OR transport*[tiab] OR opening[tiab] OR lesion[tiab]))

OR

((“blood-nerve barrier”[tiab] OR “blood nerve barrier”[tiab] OR “blood dorsal root ganglion barrier”[tiab] OR perineuri*[tiab] OR “nerve barrier”[tiab] OR (“peripheral nerve”[tiab] AND barrier*[tiab])) AND (diabet*[tiab] OR neuropath*[tiab] OR neuritis[tiab] OR autoimmun*[tiab] OR immunoglobulin*[tiab] OR IgG[tiab] OR endotheli*[tiab] OR microvessel*[tiab] OR permeab*[tiab] OR macrophage*[tiab] OR transcriptom*[tiab] OR “dorsal root ganglion”[tiab] OR structur*[tiab]))

OR

((“neurovascular unit”[tiab] OR “neurovascular barrier”[tiab] OR “neurovascular barriers”[tiab] OR “blood-brain barrier”[tiab] OR “blood brain barrier”[tiab]) AND (Parkinson*[tiab] OR “multiple system atrophy”[tiab]) AND (dysfunct*[tiab] OR permeab*[tiab] OR leak*[tiab] OR opening[tiab] OR transcyt*[tiab] OR neuroinflamm*[tiab] OR microglia*[tiab] OR “extracellular vesicle”[tiab] OR “extracellular vesicles”[tiab]))

OR

((“blood-brain barrier”[tiab] OR “blood-nerve barrier”[tiab]) AND “health and disease”[tiab])

OR

((“neurovascular unit”[tiab] OR “neurovascular barrier”[tiab] OR “neurovascular barriers”[tiab] OR “blood-brain barrier”[tiab] OR “blood brain barrier”[tiab]) AND ((overview[tiab] AND (structur*[tiab] OR function*[tiab] OR biomarker*[tiab] OR integrity[tiab])) OR “basic physiology”[tiab] OR “molecular constituent”[tiab] OR “molecular constituents”[tiab] OR (pericyt*[tiab] AND regulat*[tiab]) OR (aging[tiab] AND transcyt*[tiab]) OR (“traumatic brain injury”[tiab] AND fibrin*[tiab]) OR (“lipid transport”[tiab] AND transcyt*[tiab]) OR (neutrophil*[tiab] AND (“extracellular vesicle”[tiab] OR “extracellular vesicles”[tiab])) OR (neurodegenerat*[tiab] AND biomarker*[tiab]) OR (macrophage*[tiab] AND endotheli*[tiab]) OR (transcriptom*[tiab] AND (endotheli*[tiab] OR dysfunct*[tiab])) OR ((“tight junction”[tiab] OR “tight junctions”[tiab]) AND dysfunct*[tiab]) OR (“cerebral hypoperfusion”[tiab] AND VCAM1[tiab]) OR (“ischemic stroke”[tiab] AND occludin[tiab]) OR (“intracranial hemorrhage”[tiab] AND (“tight junction”[tiab] OR “tight junctions”[tiab]))))

OR

(((“cardiac autonomic neuropathy”[tiab] OR “cardiovascular autonomic neuropathy”[tiab] OR “cardiac autonomic dysfunction”[tiab] OR “autonomic neuropathy”[tiab]) AND (diabet*[tiab] OR “glucose tolerance”[tiab])) OR (dysautonom*[tiab] AND autoimmun*[tiab]) OR (“autonomic dysfunction”[tiab] AND ((hypertension[tiab] AND (inflamm*[tiab] OR “angiotensin II”[tiab] OR “oxidative stress”[tiab])) OR (exercise[tiab] AND (microglia*[tiab] OR “paraventricular nucleus”[tiab] OR inflamm*[tiab])))) OR (“heart rate variability”[tiab] AND (“autonomic nervous system imbalance”[tiab] OR dysautonom*[tiab])) OR (“rostral ventrolateral medulla”[tiab] AND (hypertension[tiab] OR neuroinflamm*[tiab] OR “cardiovascular dysregulation”[tiab])) OR ((“nucleus of the solitary tract”[tiab] OR “nucleus tractus solitarius”[tiab]) AND respiratory[tiab] AND sympath*[tiab]) OR (“paraventricular nucleus”[tiab] AND ((sympath*[tiab] AND hypertension[tiab]) OR (microglia*[tiab] AND “autonomic dysfunction”[tiab]) OR (exercise[tiab] AND “autonomic dysfunction”[tiab]))) OR (“autonomic dysreflexia”[tiab] AND “spinal cord injury”[tiab]) OR ((“sympathetic ganglion”[tiab] OR “sympathetic ganglia”[tiab]) AND (permeab*[tiab] OR microvasculature*[tiab] OR capillar*[tiab] OR barrier*[tiab])) OR (“autonomic ganglionopathy”[tiab] AND (autoimmun*[tiab] OR IgG[tiab])))

OR

(((“Guillain-Barre syndrome”[tiab] OR “Guillain-Barré syndrome”[tiab]) AND (“acute autoimmune neuropathies”[tiab] OR “autonomic dysfunction”[tiab])) OR (Alzheimer*[tiab] AND ((microglia*[tiab] AND complement*[tiab]) OR (fibrinogen[tiab] AND microglia*[tiab]))) OR (“multiple system atrophy”[tiab] AND (microglia*[tiab] OR (glia[tiab] AND Parkinson*[tiab])))))

AND english[la] AND 1995/01/01:2026/08/31[dp]
